# Response to vanadate exposure in *Ochrobactrum tritici* strains

**DOI:** 10.1371/journal.pone.0229359

**Published:** 2020-02-24

**Authors:** Mariana Cruz Almeida, Rita Branco, Paula V. Morais

**Affiliations:** CEMMPRE, Centre for Mechanical Engineering, Materials and Processes, Department of Life Sciences, University of Coimbra, Coimbra, Portugal; Cinvestav, MEXICO

## Abstract

Vanadium is a transition metal that has been added recently to the EU list of Raw Critical Metals. The growing needs of vanadium primarily in the steel industry justify its increasing economic value. However, because mining of vanadium sources (i. e. ores, concentrates and vanadiferous slags) is expanding, so is vanadium environmental contamination. Bioleaching comes forth as smart strategy to deal with supply demand and environmental contamination. It requires organisms that are able to mobilize the metal and at the same time are resistant to the leachate generated. Here, we investigated the molecular mechanisms underlying vanadium resistance in *Ochrobactrum tritici* strains. The highly resistant strain 5bvl1 was able to grow at concentrations > 30 mM vanadate, while the *O*. *tritici* type strain only tolerated < 3 mM vanadate concentrations. Screening of *O*. *tritici* single mutants (*chrA*, *chrC*, *chrF* and *recA*) growth during vanadate exposure revealed that vanadate resistance was associated with chromate resistance mechanisms (in particular ChrA, an efflux pump and ChrC, a superoxide dismutase). We also showed that sensitivity to vanadate was correlated with increased accumulation of vanadate intracellularly, while in resistant cells this was not found. Other up-regulated proteins found during vanadate exposure were ABC transporters for methionine and iron, suggesting that cellular responses to vanadate toxicity may also induce changes in unspecific transport and chelation of vanadate.

## Introduction

Vanadium (V) is a transition metal, which can exist in two oxidation states at neutral pH: V(IV) (vanadyl ion, cationic species VO^2^) and V(V) (vanadate ion, anionic species H_2_VO_4_) [1]. With an abundance of 0.013%, vanadium is considered a relatively abundant element on Earth and a very valuable resource for different industrial applications. In fact, 80% of all V produced worldly is being used as an additive for steel industry [2].

Vanadium primary resources come from ores, concentrates and vanadiferous slags and are mainly mined in South Africa, China, Russia, and the USA [2]. Recently, the European Union (EU) has published the third list of Critical Raw Materials where vanadium is listed as critical for the EU economy. A critical metal is defined as one whose lack of availability during a national emergency would affect the economic and defensive capabilities of that country [3].

Because vanadium is being extensively used not only for the traditional steel, automobile and aviation industries but also in the newest vanadium redox flow batteries, its supply demands will undoubtedly increase further. New technologies are welcome to recover and recirculate vanadium from materials at the end of use. Bioleaching is an accepted strategy that requires organisms able to mobilize the metal and at the same, time resistant to the leachate generated. Besides, the use of organisms able to accumulate vanadium, either intracellularly or on the surface of cell membranes, could enhance the vanadium recovery process.

During the last two decades, microbial metal accumulation has received increased attention due to the potential use of microbes as “metal living factories”, for metal uptake, transformation and accumulation [4, 5]. Metal accumulation can occur in two ways: i) by metabolism-independent passive sorption; ii) by metabolism-dependent active uptake. This energy-driven process dependent on active metabolism normally occurs when an element is absorbed in a faster rate and then retained by an organism (bioaccumulation). Intracellular accumulation is especially important for essential metals. However, non-essential metals can also be taken up due to similar chemistry to essential ones [4].

Vanadium toxicity is comparable to that of lead, mercury and arsenic [6]. Nevertheless, V(III), V(IV) and V(V) species are of biological relevance and nature has evolved several enzymatic systems using vanadium in their active sites as relevant components for their function including vanadium-dependent haloperoxidases, nitrogenases and vanabins [7]. On the other hand, by mimicking the phosphate ion (PO_4_^3-^), vanadate (VO_4_^3-^) can form transition-state analogues of phospho-protein intermediaries and therefore can easily substitute for phosphate in enzymes such as phosphatases and kinases [8].

A restricted number of bacteria can employ vanadium in varying biological functions. Vanadium nitrogenases have been reported in bacterial strains belonging to the genus *Azotobacter* and in cyanobacteria of the genera *Anabaena* and *Nostoc* [9, 10]. Vanadate-dependent haloperoxidases have been found in bacteria such as *Streptomyces* and marine cyanobacteria [11]. Several studies have reported that bacteria of the genera *Shewanella*, *Pseudomonas* and *Geobacter* can use vanadate as a primary electron acceptor in respiration [12–15]. Moreover, vanadium oxidation forms as vanadate and vanadyl ions can complex with carboxylate, catecholate and hydroxamate ligands present in siderophores [16].

Vanadium resistant microorganisms have been isolated from environmental contaminated sites such as phytoremediation plants (up to 30 mM Na_3_VO_4_), V mining-impacted soils [17] and the intestine of the vanadium-rich ascidian *Ascidia sydneiensis samea* (10 mM Na_3_VO_4_) [18–20]. However, the molecular mechanisms underlying vanadium resistance in microorganisms are still poorly understood. Only a study in *P*. *aeruginosa* described the importance of an efflux pump, MexGHI-OpmD, for the bacterial resistance to vanadium [21]. However, it is not known whether this is a direct or indirect effect since this pump exerts other cellular roles [21, 22].

This work aimed to determine whether vanadate resistance in *Ochrobactrum tritici* strains is related to low uptake of the metal or due to the presence of efflux systems (or both). Considering the chemical structure vanadate (VO_4_^3-^) and its similarity to arsenate (AsO_4_^3-^), it could be supposed that bacteria could use arsenate-detoxifying mechanisms to detoxify vanadate. To examine this possibility, two strains of *Ochrobactrum tritici*, the type strain SCII24^T^ (resistant to both arsenate and arsenite) and 5bvl1 (less resistant to arsenate and sensitive to arsenite) were used to test the vanadate resistance. Notably, the assays to define the tolerance to vanadate by the arsenite and arsenate resistant strain SCII24^T^ showed a vanadate sensitive profile, which questioning the involvement of the arsenite/arsenate resistance genetic determinants in vanadate resistance. Considering that vanadium and chromium are transition metals, neighbors of the fourth period of the periodic table, in this work, we hypothesize that vanadate resistance mechanism could be correlated with chromate resistance mechanisms (in particular ChrA efflux pump). Therefore, the bacterial sensitivity to vanadate could be due to accumulation of vanadate intracellularly, while resistant cells would possess a vanadate export mechanism as a detoxification strategy.

## Materials and methods

### Media and growth conditions

Bacterial strains used in this study are listed in Table 1, and their resistance to chromate, arsenate and arsenite, already studied in previous works are specified. Strain 5bvl1 was previously isolated from activated sludge in a chromium-contaminated area [23], while type strain SCII24^T^ was isolated from rhizosphere of wheat [24].

**Table 1:** *Ochrobactrum tritici* strains used in this work.

*Strain name*	*Relevant characteristics*	*Reference*
*Type strain SCII24^T^*	Type strain; Cr(VI)^S^; As(III)^R^	LMG18957
*5bvl1*	Wild type; Cr(VI)^R^; As(III)^S^	[25]
*E117*	Mutant of 5bvl1; Tn*5* inserted into *chrA* gene; Cr(VI)^S^	[26]
*E117:chrA*	Mutant of 5bvl1 complemented with the *chrA* gene cloned in pBBR1MCS-5 vector; Cr(VI)^R^	[26]
*recA*	Single mutant of 5bvl1; *recA* mutated	Unpublished work
*chrCF*	Double mutant of 5bvl1; c*hrC* and *chrF* mutated	[26]
*chrC*	Single mutant of 5bvl1; *chrC* mutated	[26]
*chrF*	Single mutant of 5bvl1; *chrF* mutated	[26]

*O*. *tritici* strains were grown aerobically at 30 °C in R2A medium. The final pH of the medium was 7.2. Gentamicin (15 μg/ml) was included for selection of mutant strains when mobilizing plasmids into *O*. *tritici* strain 5bvl1 [25, 26]. Na_3_VO_4_ (Alfa Aesar, Thermo Scientific) was used to prepare 0.25 M vanadate stock solutions, which were sterilized by filtration.

### Minimum inhibitory concentration (MIC) assays

Minimum inhibitory concentration (MIC) determination was performed in solid R2A plates. Briefly, 20 μl of cellular suspensions (10^8^ CFU/ml), prepared in NaCl 0.9%, were placed onto agar R2A medium plates containing increased concentrations of vanadate (0, 3, 7.5, 15 and 30 mM) and incubated for 24 h at 30 °C (until growth was observed in control plate with no metal). Each assay was repeated in triplicate to ensure reproducibility of the experiments. The MIC was the lowest concentration of vanadate that completely inhibited bacterial growth.

To evaluate inhibition of growth strains SCII24^T^, 5bvl1, E117 and E117:*chrA* mutants were grown at 30 °C with shaking in liquid R2A medium supplemented with several concentrations of vanadate (0, 15 and 30 mM).

### Vanadate intracellular accumulation

#### Vanadate induction

Overnight cultures were diluted in 50 ml of the new R2A liquid medium and grown to exponential phase. Accumulation assays were initiated by adding different vanadate concentrations (3 and 15 mM) to 0.7 OD_600_ exponential phase cultures, followed by incubation at 30 °C with shaking for 3 h. Control and V(V)-exposed cells were harvested by centrifugation and washed twice with cold phosphate-buffered saline (PBS). Cellular lysis was performed by acid treatment at 50 °C for 1h (10% HNO_3_). Total vanadium was analyzed by Inductively Coupled Plasma Mass Spectrometry (ICP-MS) in an ICP-MS Thermo X Series, and total protein was determined using the Bradford assay [27]. Intracellular vanadium content was expressed as nanogram of V per microgram of total cellular protein.

#### Vanadate and chromate induction

To test whether activation of the Tn*OtChr* operon would influence vanadate accumulation by *O*. *tritici* strains, the same experimental procedure described above for vanadate induction was used, except that 100 μM chromate was added simultaneously with vanadate.

#### Vanadate induction during cell growth

Overnight cultures of *O*. *tritici* SCII24^T^, 5bvl1, E117 and E117:*chrA* mutants strains were diluted in 50 ml of the new R2A liquid medium to 0.06 OD_600_ and vanadate was added at 0 and 15 mM. Cells were grown for 6, 24 and 48 h, harvested, washed and acid-washed by the same procedure described above.

### Protein profiles during V accumulation

From accumulation experiments described above, a cellular pellet resuspended in PBS was lysed by sonication, using a sonicator Vibra cell (Sonics & Materials, Inc, USA) for 4 cycles of 30 seconds (with intervals of 30 seconds on ice). Lysates were centrifuged at 13,000 rpm for 15 min at 4 °C. Supernatant fractions were kept on ice until run in SDS-PAGE gels. Total protein extracts (20 μg of protein) were run in a 5–12% acrylamide gel for 60 min at 180V in running buffer (5 mM Tris-HCl pH 8.8, 0.2 M glycine, 3.5 mM sodium dodecyl sulfate (SDS)), followed by coomassie blue staining for 30 min at room temperature and then, destaining overnight. Protein profiles were compared with control and the selected protein bands (overexpressed bands) were cut from the SDS-PAGE gel and identified by LC-MS Orbitrap at the IPATIMUP Proteomics Facility.

### Statistical analysis

Prism 6 (GraphPad) using a one-way analysis of variance (ANOVA) followed by Tuckey’s multiple comparison test analysis was used to compare between strains and unpaired Student *t* test when appropriate. All results are presented as mean ± SD with n = 3, unless stated otherwise. A p value of 0.05 or less was considered significant.

## Results

### Vanadate resistance of *O*. *tritici* strains

Previous studies have characterized the resistance of *O*. *tritici* type strain SCII24^T^ and 5bvl1 strain to arsenite/arsenate (As III/V) and chromate (Cr VI), respectively [26, 28]. These results prompt us to question whether arsenite and arsenate-resistant *O*. *tritici* type strain SCII24^T^ would also be resistant to vanadate (Na_3_VO_4_).

**Fig 1:**
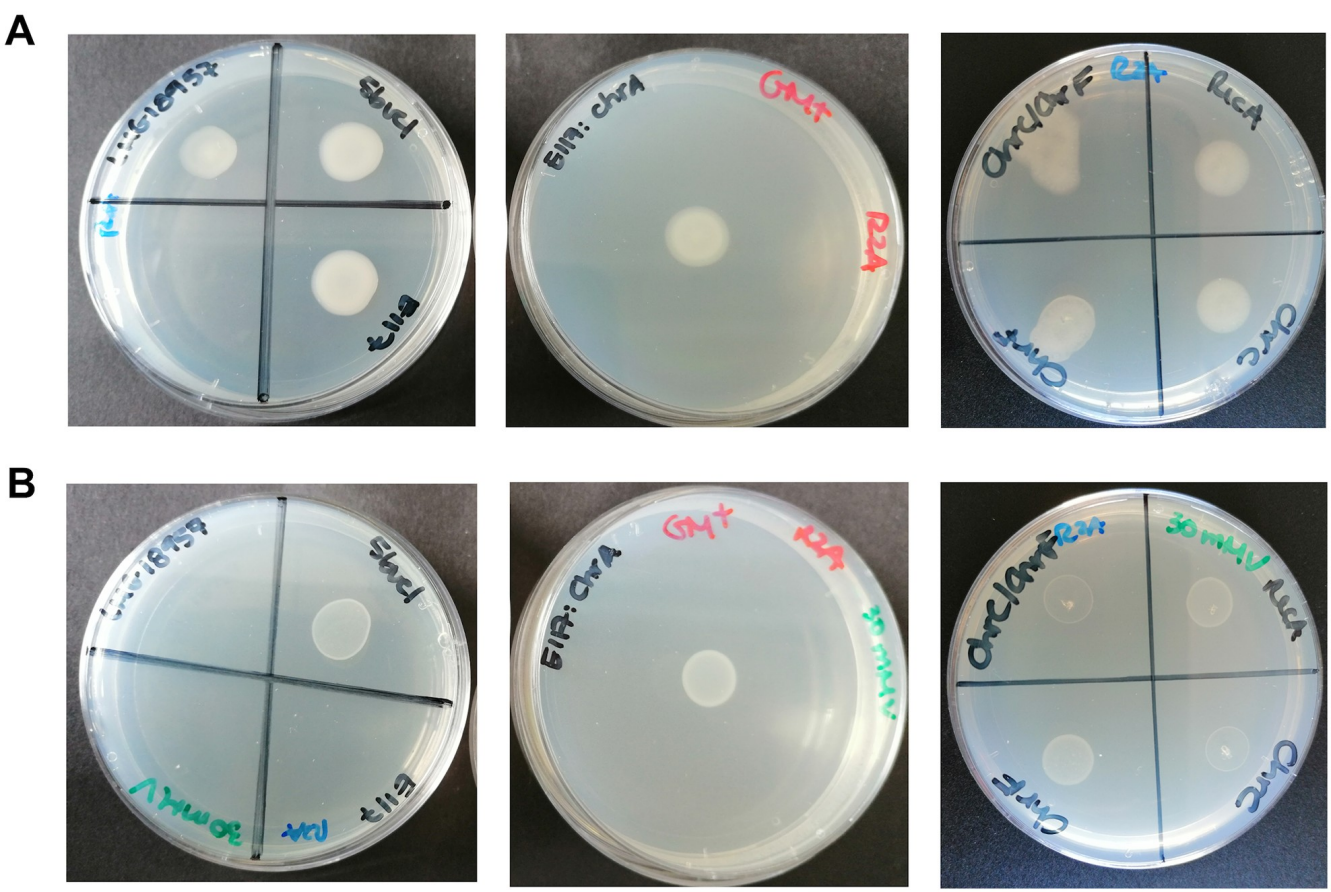
Growth of *O*. *tritici* SCII24^T^, 5bvl1, E117, E117:*chrA*, *recA*, *chrC*, *chrF* and *chrC/chrF* mutant strains on R2A plates. (A) or containing 30 mM vanadate (B) for three independent experiments (n = 3).

Vanadate concentrations from 0 to 30 mM were tested in solid medium and MIC was determinate (Fig 1 and Table 2).

**Table 2:** *O*. *tritici* strains vanadate MIC in solid R2A medium.

*Strain name*	*Relevant characteristics*	*MIC* *V (mM)*	*Vanadate resistance*
*SCII24^T^*	Type strain; Cr(VI)s; As(III)^R^	3	Sensitive
*5bvl1*	Wild type; Cr(VI)^R^; As(III)^S^	> 30	Resistant
*E117*	Mutant of 5bvl1; Tn*5* inserted into *chrA* gene; Cr(VI)s	7.5	Sensitive
*E117:chrA*	Mutant of 5bvl1 complemented with the *chrA* gene cloned in pBBR1MCS-5 vector Cr(VI)^R^	> 30	Resistant
*recA*	Single mutant of 5bvl1; *recA* mutated	> 30	Resistant
*chrCF*	Double mutant of 5bvl1; c*hrC* and *chrF* mutated	30	Resistant
*chrC*	Single mutant of 5bvl1; *chrC* mutated	30	Resistant
*chrF*	Single mutant of 5bvl1; *chrF* mutated	> 30	Resistant

The strain *O*. *tritici* SCII24^T^ (Cr(VI)^S^; As(III)^R^) was clearly sensitive to vanadate (MIC at 3 mM vanadate), while strain 5bvl1 (Cr(VI)^R^; As(III)^S^) was resistant (MIC > 30 mM vanadate). To validate that the V-resistance could be related with the Cr (VI) resistance mechanism, involving the ChrBACF system, the ability of 5bvl1 mutants (strains E117, E117:*chrA*, *chrC*, *chrF*, *chrCF*) to grow in R2A plates containing up to 30 mM vanadate (Fig 1) was evaluated. As it is recognized that RecA protein essential for the repair and maintenance of the DNA has a relevant role as a protective protein to general stress, a *recA* mutant was also tested (unpublished work).

The results showed that the E117 mutant (where *chrA* gene is mutated in the 5bvl1 strain background) was only able to grow up to 7.5 mM vanadate. This vanadate resistance profile is similar to what was found for the type strain SCII24^T^, and very different from strain 5bl1, revealing the mutant E117 as sensitive to vanadate. When the integrity of *chrA* gene in the E117 mutant was reestablished by complementation (strain E117:*chrA*), V(V)-resistance to more than 30 mM vanadate was restored, suggesting that ChrA protein is involved in the vanadate resistance mechanism of this strain.

Mutant strains *recA*, *chrF* were resistant to vanadate (MIC > 30 mM vanadate, similarly to 5bvl1 and E117c*ChrA*) while *chrC* and *chrCF* exhibited decreased growth ability at 30 mM vanadate (MIC = 30 mM vanadate).

**Fig 2:**
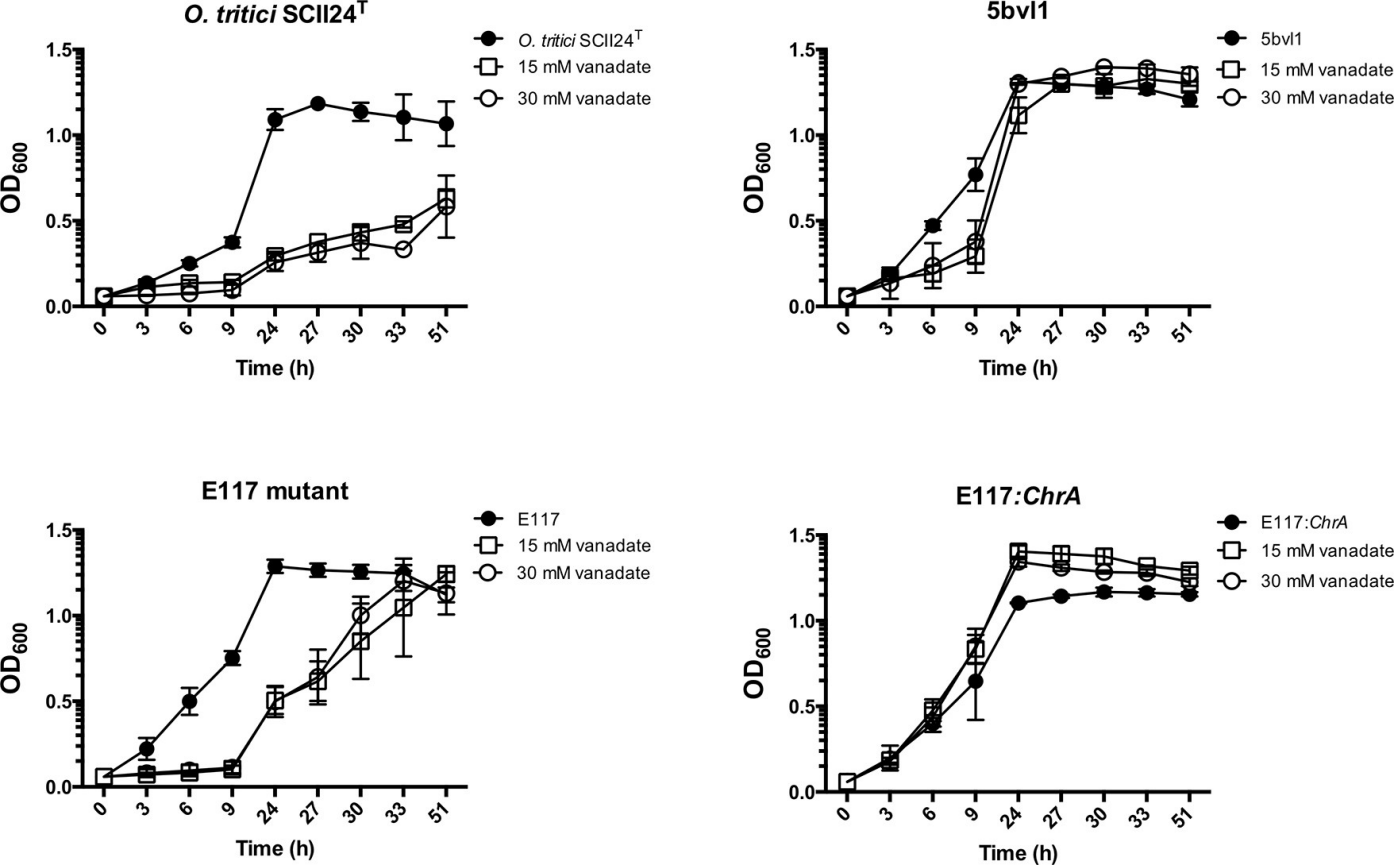
Sensitivity of *O*. *tritici* strains to vanadate. Cultures of *O*. *tritici* type strain SCII24^T^, 5bvl1, E117, E117:*chrA* mutant strains were grown in R2A liquid medium for 51 h at 30 °C in the presence of the indicated concentrations of vanadate (mean ± SD, n = 3).

To validate the vanadate resistance profile of *O*. *tritici* strains (SCII24^T^, E117 mutant, 5bvl1 and E117:*chrA* mutant) to vanadate, their ability to grow in the absence or presence of 15 and 30 mM vanadate in liquid R2A medium was evaluated. Growth curves were recorded up to 51 h at 30 °C with shaking (Fig 2).

Similarly, to the plate resistance assays, in liquid R2A broth at 24 h (Fig 2), *O*. *tritici* SCII24^T^ and E117 mutant, both showed decreased growth when vanadate was added compared with R2A medium without vanadate. When the growth period was prolonged, mutant strain E117 achieved similar maximum OD_600_ as when grown in R2A liquid medium without vanadate. Contrarily, 5bvl1 and E117:*chrA* mutant strains did not show any difference in growth when vanadate was present (at 15 and 30 mM), confirming that these strains are resistant to V(V).

### Intracellular vanadate accumulation

**Fig 3:**
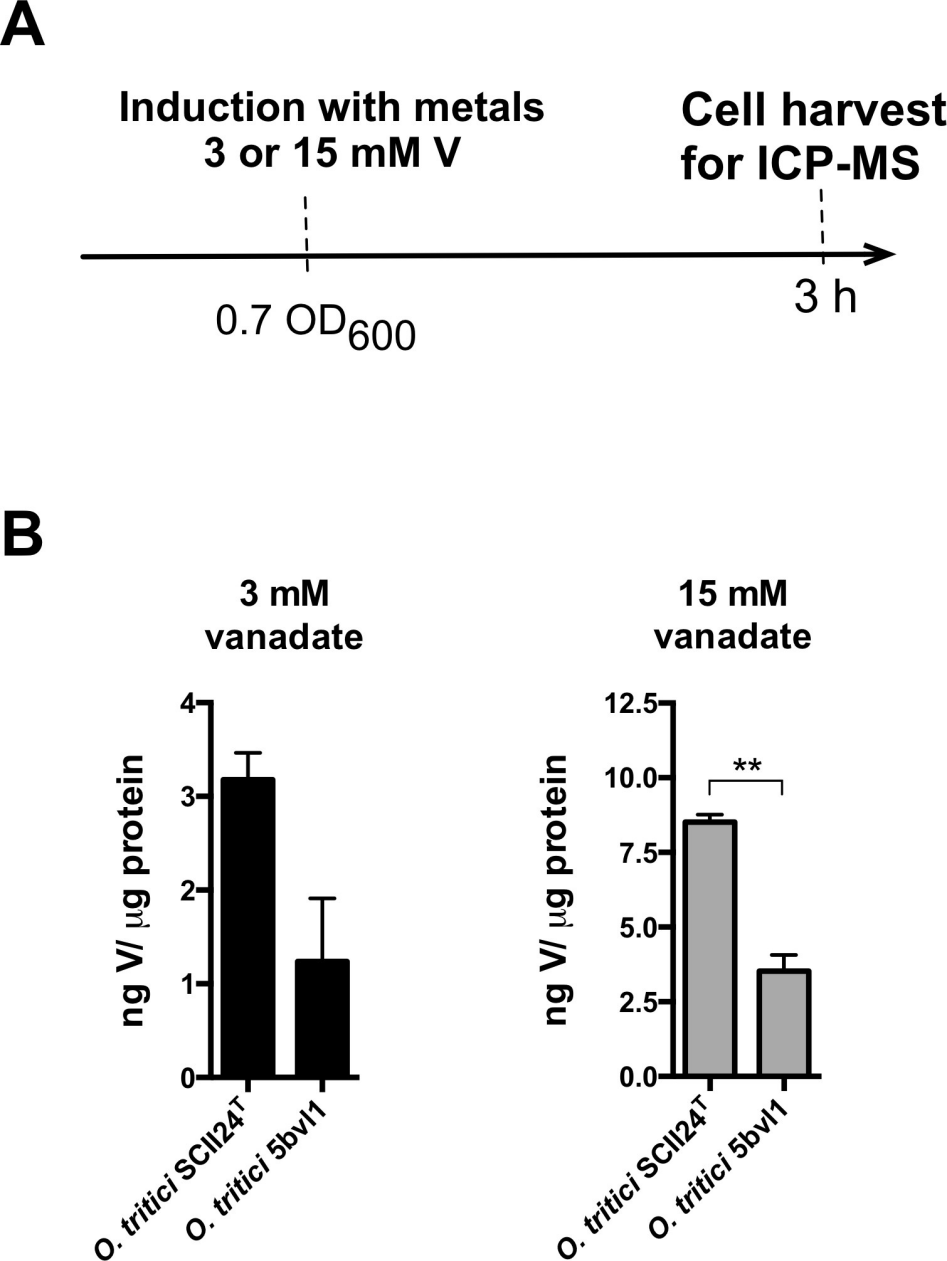
Vanadate intracellular accumulation by *O*. *tritici* SCII24^T^ and 5bvl1 strains after 3 or 15 mM vanadate induction for 3 h at 30 °C. Results are the intracellular concentrations of V after normalization for protein content (mean ± SD, n = 2) **, p < 0.01.

To determine if vanadate toxicity in *O*. *tritici* SCII24^T^ and E117 mutant strains were due to cells accumulating vanadate intracellularly, SCII24^T^ (sensitive) and 5bvl1 (resistant) strains were grown in liquid R2A broth medium until 0.7 OD_600_ was reached. Vanadate (3 mM, in which sensitive strains still grow and 15 mM, where only resistant strains grow) was added and cells were allowed to grow at 30 °C with shaking for 3 h (Fig 3A). In the presence of 3 mM vanadate (Fig 3B), cells showed increased vanadate concentration intracellularly in the sensitive type strain SCII24^T^ while the resistant 5bvl1 showed lower levels of intracellular vanadate. With 15 mM vanadate exposure, this trend was more significant, with resistant strain 5bvl1 accumulating only 3.5 ± 0.38 ng V/μg protein and type strain SCII24^T^ accumulating 8.5 ± 0.17 ng V/μg protein (approximately 2.5 fold increase, p < 0.01).

**Fig 4:**
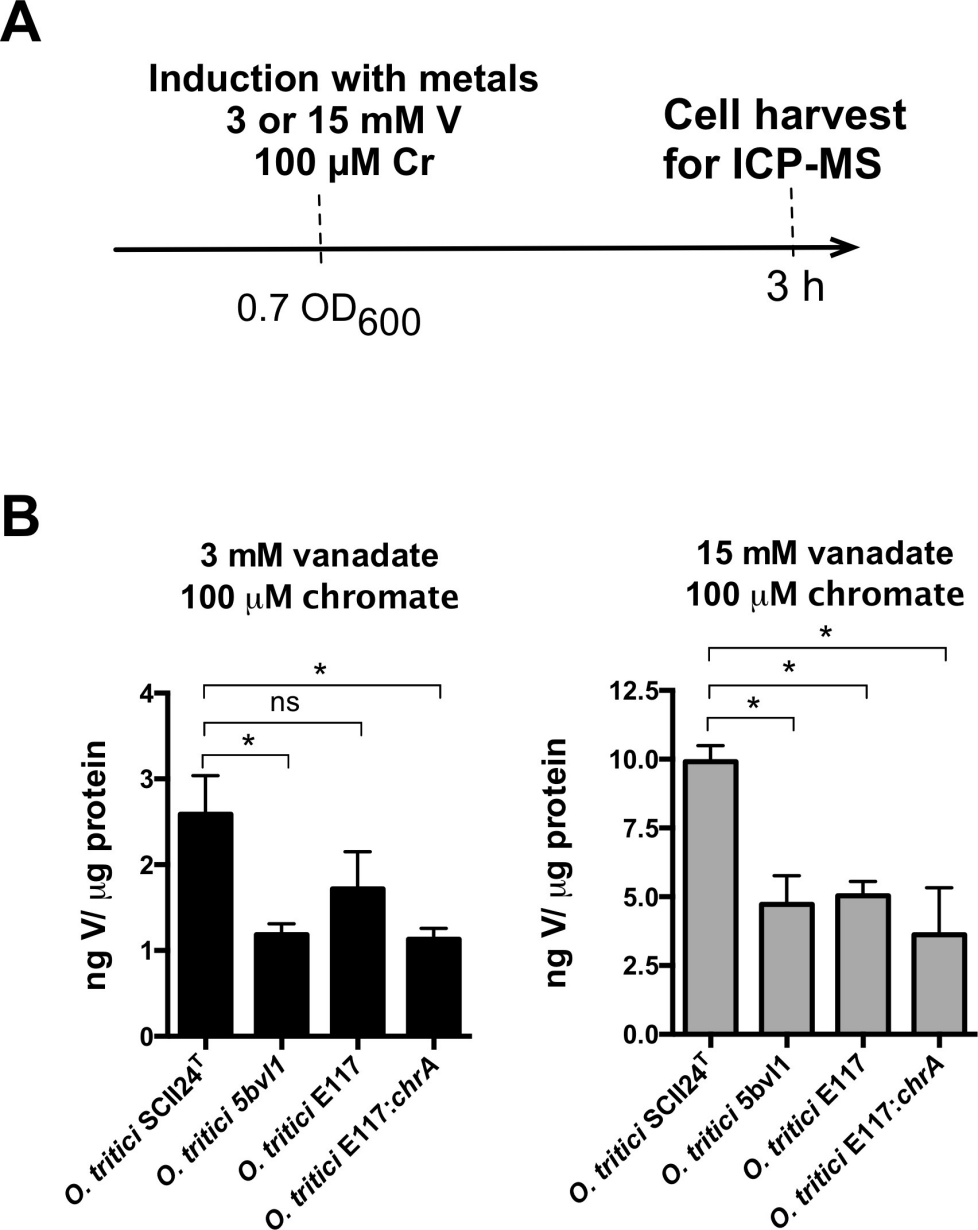
Vanadate intracellular accumulation by *O*. *tritici* SCII24^T^, 5bvl1, E117, E117:*chrA* strains after vanadate (3 or 15 mM) and chromate (100 μM) induction for 3 h at 30 °C. Results are the intracellular concentrations of V after normalization for protein content (mean ± SD, n = 2) ns, not significant; *, p < 0.05.

Due to decreased intracellular accumulation of this metal in strain 5bvl1, we further considered a possible relation between the *chr* operon induction by Cr(VI) and the decrease of the amount of vanadate accumulation. Both vanadate sensitive (SCII24^T^ and E117) and resistant (5bvl1 and E117:*chrA*) cells induced simultaneously with 3 mM vanadate and 100 μM chromate (lowest concentration required for induction of Tn*Otchr* [26]), showed the same vanadate accumulation as without Cr(VI) induction (Figs 3B and 4B). Similarly, cells induced simultaneously with 15 mM vanadate and 100 μM chromate also showed the same accumulation of vanadate without C(VI)-induction (Figs 3B and 4B), suggesting that the activation of the chromium resistance *chr* operon is not directly involved in the amount of vanadate accumulated by the cells. Mutant strain E117, did not show the same vanadate accumulation at 15 mM V(V) as type strain SCII24^T^ (Fig 4B). This can be explained as a consequence of the genetic manipulation to disrupt *chrA* by transposon introduction, which may have consequently cause a frameshift in the other *chr* genes within the Tn*Otchr* operon (involved in stress control). Moreover, increasing 5 times the amount of vanadate added to cells (15 mM) also showed a 5-fold increase in intracellular vanadate for each strain (Fig 4B), similarly to what was found for cells induced only with vanadate (Fig 3B).

### Protein profiles during vanadate exposure

**Fig 5:**
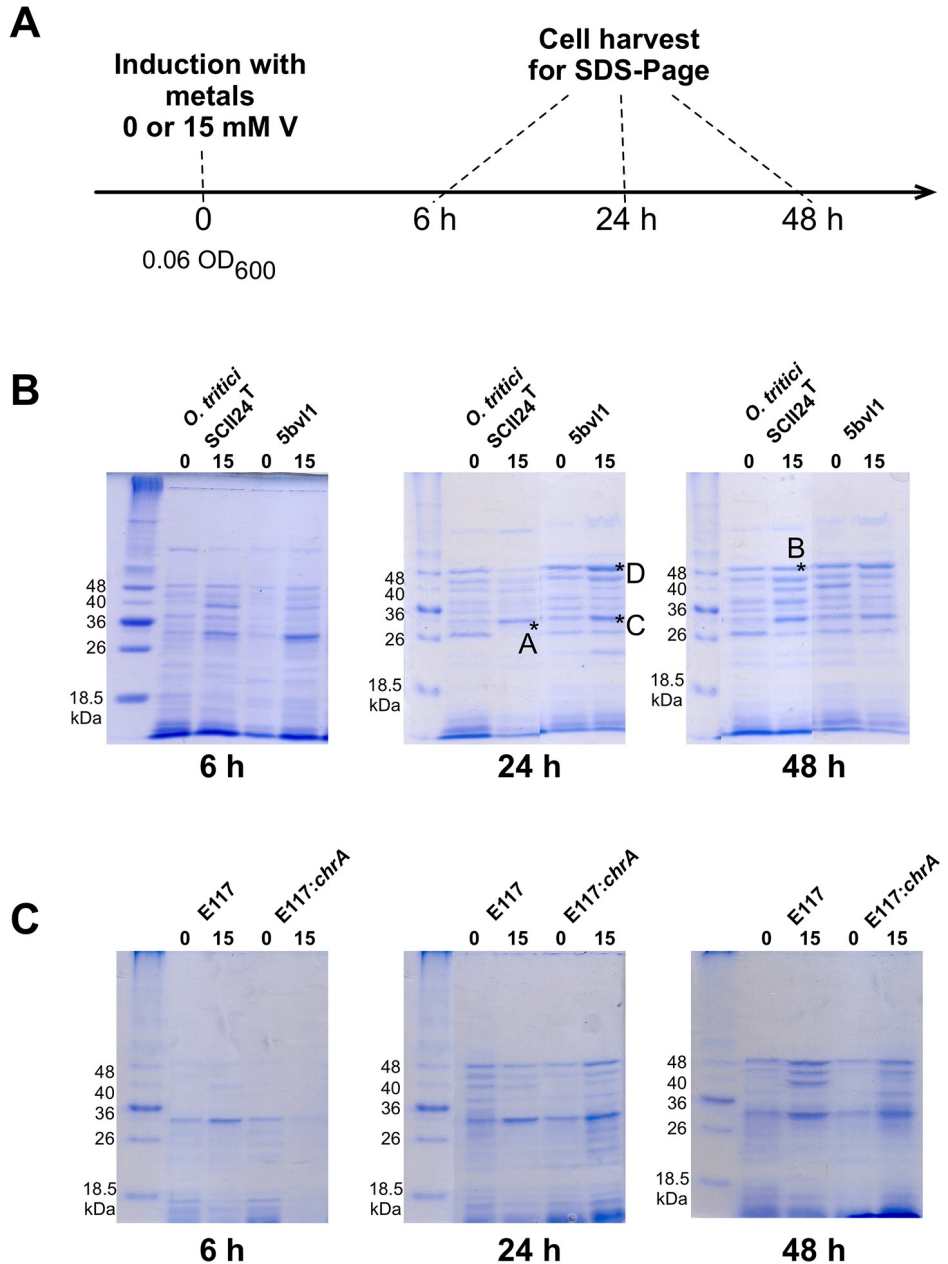
Total normalized protein profiles of *O*. *tritici* strains. (A) *O*. *tritici* SCII24^T^, 5bvl1, (B) E117 and E117:*chrA* strains after 6, 24 and 48 h growth at 30 °C in the absence or presence of 15 mM vanadate. *A, *B, *C and *D, represent bands cut for identification by LC-MS Orbitrap. Gels were grouped by incubation time.

In order to determine potential protein targets required for vanadate detoxification in V-resistant strains *O*. *tritici* 5bvl1 and E117:*chrA*, cells were grown with or without 15 mM vanadate and total protein extracts were obtained. SDS-PAGE gels were run and strain-specific protein profiles were compared between sensitive (SCII24^T^ and E117) and resistant strains (5bvl1 and E117:*chrA*), and between in absence or presence of vanadate at each time point (Fig 5).

The *O*. *tritici* SCII24^T^ showed at 24 h a similar profile to the V(V)-resistant 5bvl1 strain at 6h and likewise, at 48 h, sensitive strain SCII24^T^ displayed a similar protein profile to resistant 5bvl1 strain at 24 h. This result suggests that sensitivity of *O*. *tritici* SCII24^T^ may be due to a slower efficient response to vanadate ions toxicity, which seems to be delayed compared with resistant strain 5bvl1.

Likewise, the sensitive E117 mutant strain shows an identical protein profile at 6 and 24 h with vanadate (similarly to what was found for type strain SCII24^T^). E117:*chrA* resistant mutant strain showed differences from 6 to 24 and from 24 to 48 h after vanadate induction, suggesting that this mutant strain, like 5bvl1, responds to vanadate exposure by altering its protein synthesis.

To further understand and identify other key players in vanadate detoxification responses, 2 bands at 30 kDa (bands A and C in Fig 5B) and 2 bands at 46 kDa (band B and D in Fig 5B) were cut and identified by LC-MS Orbitrap, using the available UniProt database within the taxonomic genera *Ochrobactrum* to BLAST the sequences (Table 3).

**Table 3:** Identification of protein bands by LC-MS and BLAST analysis.

*Strain*	*Band*	*Accession number*	*Identification*	*Score*	*Coverage (%)*	*Peptides*	*BLAST*
*SCII24^T^*	A	A0A011VJ37	Lipoprotein	18,44	44	10	Methionine ABC Transporter
*5bvl1*	C	A0A2P9HPU8	Lipoprotein	71,23	57	13	
*SCII24^T^*	B	A0A137XN14	Sugar ABC transporter substrate-binding protein	178,12	58	23	ABC Transporter substrate-binding protein for maltose/G3P/polyamine/iron
*5bvl1*	D	A0A137XN14	Sugar ABC transporter substrate-binding protein	1133	79	35	

Protein sequences were selected for the highest score and retrieved using the respective accession numbers. BLASTP 2.2.26 was used against the genomes of each *O*. *tritici* strains (SCII24^T^ and 5bvl1 in RAST [29]). The 30 kDa bands (A and C) were identified as a Methionine ABC Transporter (identity 98%; e value of 0.0; score of 557) and the 46 kDa bands (B and D) as an ABC Transporter, substrate-binding protein for maltose/G3P/polyamine/iron (identity 100%; e value of 0.0; score 860).

## Discussion

*Ochrobactrum tritici* strains are good models for studying the metal resistance mechanisms in bacteria. Previous works identified the chromate resistant determinant in strain *O*. *tritici* 5bvl1 coded by the *chr* operon included into the transposon, Tn*Otchr*. In the type strain SCII24^T^ the resistance to arsenic was correlated with the presence of different *ars* operons in the genome [28].

Since strain *O*. *tritici* 5bvl1 was resistant to vanadate and the type strain was sensitive, several mutants of strain 5bvl1, constructed in previous works, were explored in an attempt to understand the vanadate resistance. Since strain 5bvl1 carries the Tn*Otchr* genetic resistance system to chromate, the first question was to determine if ChrA protein (efflux pump) could be involved in the resistance profile of this strain. The results suggest that ChrA pump may play a role in vanadate resistance, since strains 5bvl1 and E117:*chrA*, both with intact *chrA* gene, show resistance to vanadate, opposite to the type strain and the mutant E117, which do not have *chrA* and are sensitive to vanadate. Nevertheless, the vanadate sensitive profiles of *chrA*-mutant and type strains were different, 7.5 mM and 3 mM, respectively. This finding suggests other possible mechanisms involved in vanadate resistance in this strain. The other genes comprised in the *chr* operon (*chrC* and *chrF*), code for two superoxide dismutase, which seem to slightly contribute to vanadate resistance, specially ChrC, similarly to what was seen in chromate treated cells [30]. The SOD-like ChrC from strain 5bvl1 shows high similarity to a Fe-SOD identified in *Cupriavidus metallidurans*, while ChrF belongs to the Mn-SOD family [31], explaining why they show different characteristics, functionalities, and metal responses. Thus, it is probable that intracellular vanadate result in reactive oxygen species (ROS) that are controlled by these additional superoxide dismutase. In fact, *P*. *aeruginosa* mutants deficient in the production of a SodB (FeSOD) have already been reported as sensitive to vanadium, while mutant deficient for Mn-SOD (SodA) were only slightly affected [32]. Therefore, FeSODs seem be extremely important in the process of superoxides detoxification generated by vanadium in V-resistant cells.

Resistance to different toxics has been described for several bacterial species and is often associated with the expression of efflux pump systems. Diverse types of pumps are involved in heavy-metal resistance [33, 34]. However, in many cases, the expression of these systems still is not completely clarified.

Efflux mechanisms for chromate and arsenite were recognized in different strains of the species *O*. *tritici*. The ability to extrude vanadate seems to be part of the resistance mechanism in *O*. *tritici* 5bvl1 since the amount of vanadium accumulated by the sensitive type strain is significantly higher. Nevertheless, extrusion through ChrA pump is not the only efflux system for vanadate in strain 5bvl1 considering the similar vanadium accumulation in V-sensitive and resistant 5bvl1 cells (native 5bvl1, E117 and E117:*chrA*). Even if vanadate could not ligate the regulator ChrB, and therefore not induce the expression of the *chr* operon, the induction with chromate assays did not show a significant change in the amount of vanadium accumulated by the cells.

The analysis of the protein expression visualized on SDS-PAGE gels supports the existence of several mechanisms activated for vanadium resistance in strain 5bvl1. The sensitive *O*. *tritici* type strain and E117 strain overexpressed, first, a Methionine ABC efflux system, and second, an ABC transporter able to transport different substrates including iron. Later on, other visible proteins could be differently recognized. This difference in profiles may be related with the accumulation of metal inside the sensitive strains, creating toxicity, inducing the transcription of proteins involved in stress control. The ABC transporters overexpressed may be involved in the transport of substrates needed by the cell under stress or may even transport vanadium. Other roles in cell homeostasis have been suggested for the Methionine ABC Transporter in *Streptococcus suis* during oxidative stress [35]. The maltose/G3P/polyamine/iron ABC transporter has been described in *Pseudomonas aeruginosa* as a transport system involved in inorganic ion transport and metabolism (NCBI Accession number WP_003091922).

It is well known that many metals are mutagenic to bacteria, affecting the nucleic acids [27]. In this sense, efficient mechanisms for DNA repairing are essential to allow bacterial coping with metals. Under metal stress conditions leading to DNA damage, the RecA protein is normally activated as coprotease, which facilitates the self-cleavage reaction of LexA [36]. Therefore, RecA is a recognized protein essential for the repair and maintenance of DNA. In this work, the strain 5bvl1 mutated at the *recA* gene was tested to evaluate the potential mutagenic effect of vanadate in *O*. *tritici* cells. The vanadate resistance of this mutated strain was not affected by vanadate, which seems to indicate that vanadate may not target drastically the bacterial DNA SOS pathway. This finding is according to the current literature since the scarce available data suggests that vanadium compounds do not produce gene mutations in standard *in vitro* tests in bacterial or mammalian cells [37].

In conclusion, *O*. *tritici* 5bvl1 strain was found to grow at high concentrations of vanadate (> 30 mM), while strain SCII24^T^ only tolerated 3 mM of vanadate. Moreover, ChrA and ChrC (a chromate efflux pump and a superoxide dismutase, respectively) were found to be important in vanadate resistance mechanisms as these single mutant strains only tolerated lower concentrations of vanadate compared with native strain 5bvl1. Vanadate cellular sensitivity was associated with increased accumulation of the metal inside bacterial cells, while in resistant cells lower accumulation was observed. Furthermore, ABC transporters for methionine and iron were found to be up-regulated in response to vanadate in both sensitive and resistant strains, but at different time points, suggesting that vanadate toxicity induces changes in transport and chelation cell systems that respond to keep cell homeostasis.

## Supporting information

S1 Fig (TIFF)

S2 Fig (TIFF)

S1 Raw images SDS-PAGE images were scanned in a routine scanner. (TIFF)
