# Supplementary figures and images for: Response to vanadate exposure in Ochrobactrum tritici strains
Source: PLoS One. 2020 Feb 24;15(2):e0229359. doi: 10.1371/journal.pone.0229359 (PMC7039435; doi:10.1371/journal.pone.0229359)

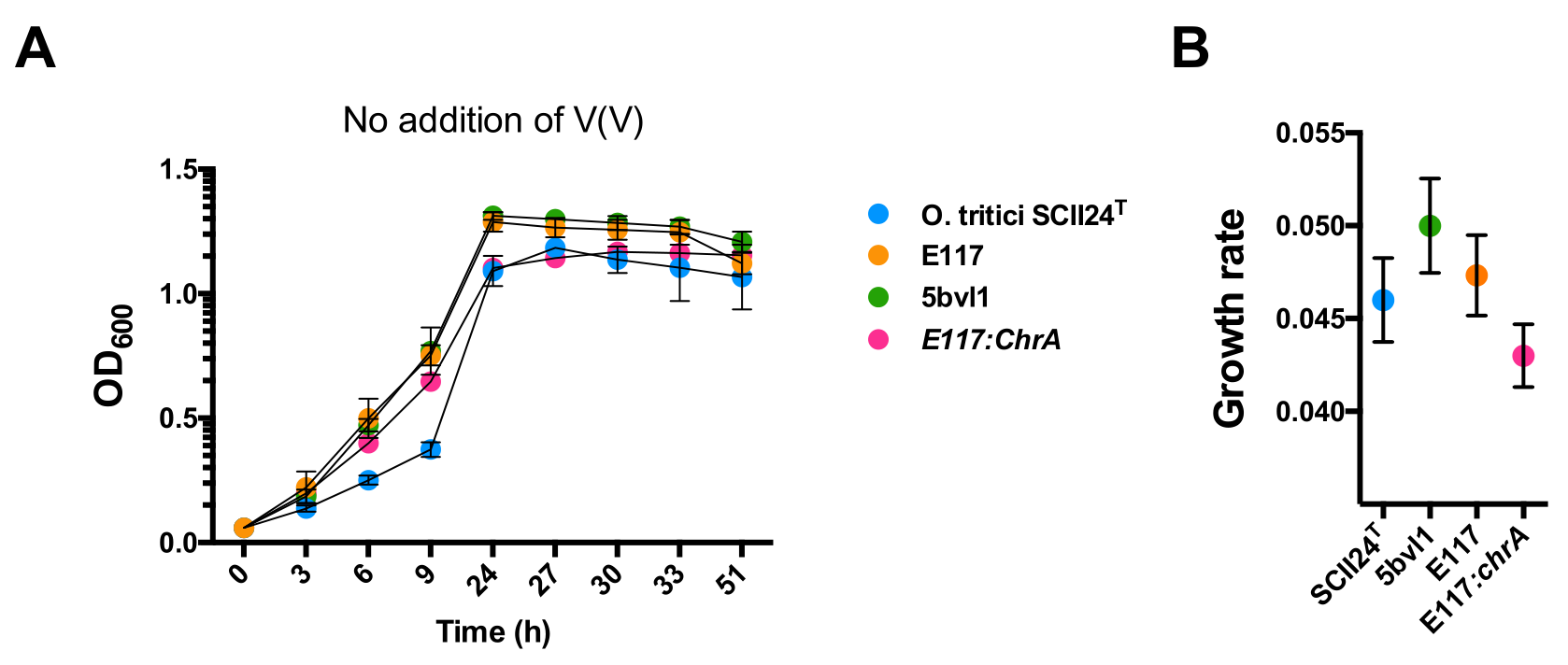

Supplement: S1 Fig — (TIFF) [file pone.0229359.s001.tiff]

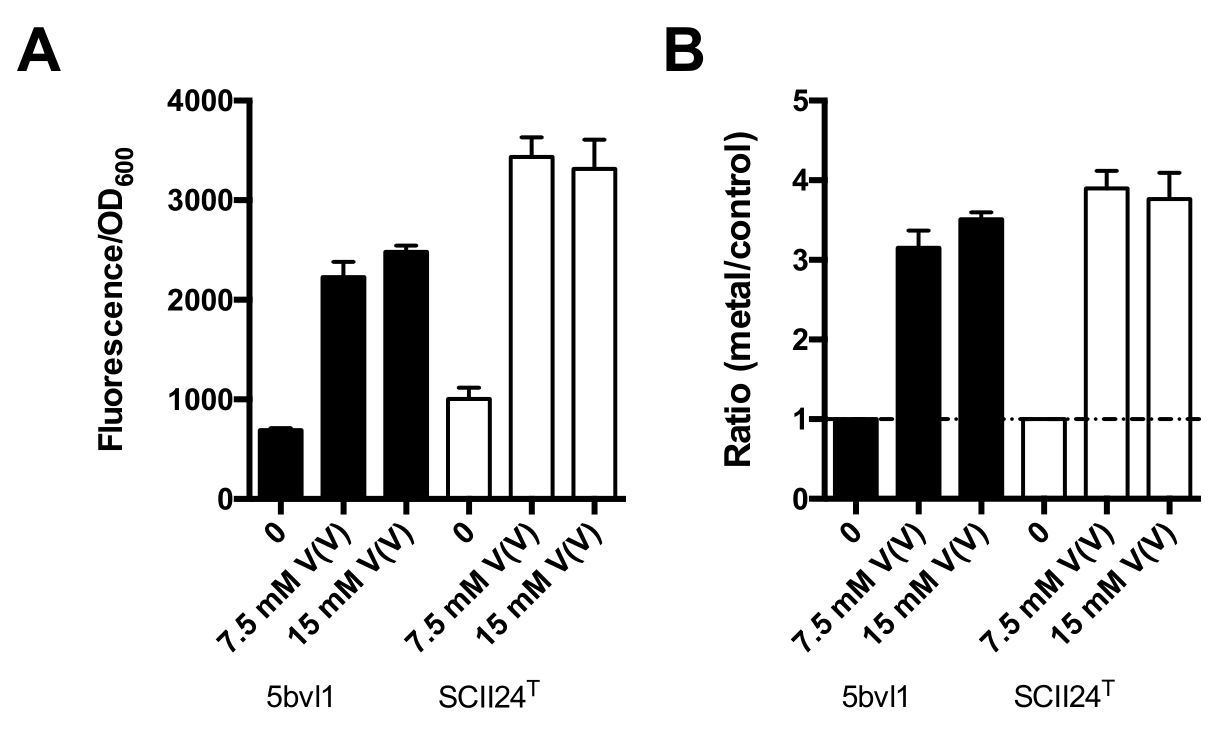

Supplement: S2 Fig — (TIFF) [file pone.0229359.s002.tiff]

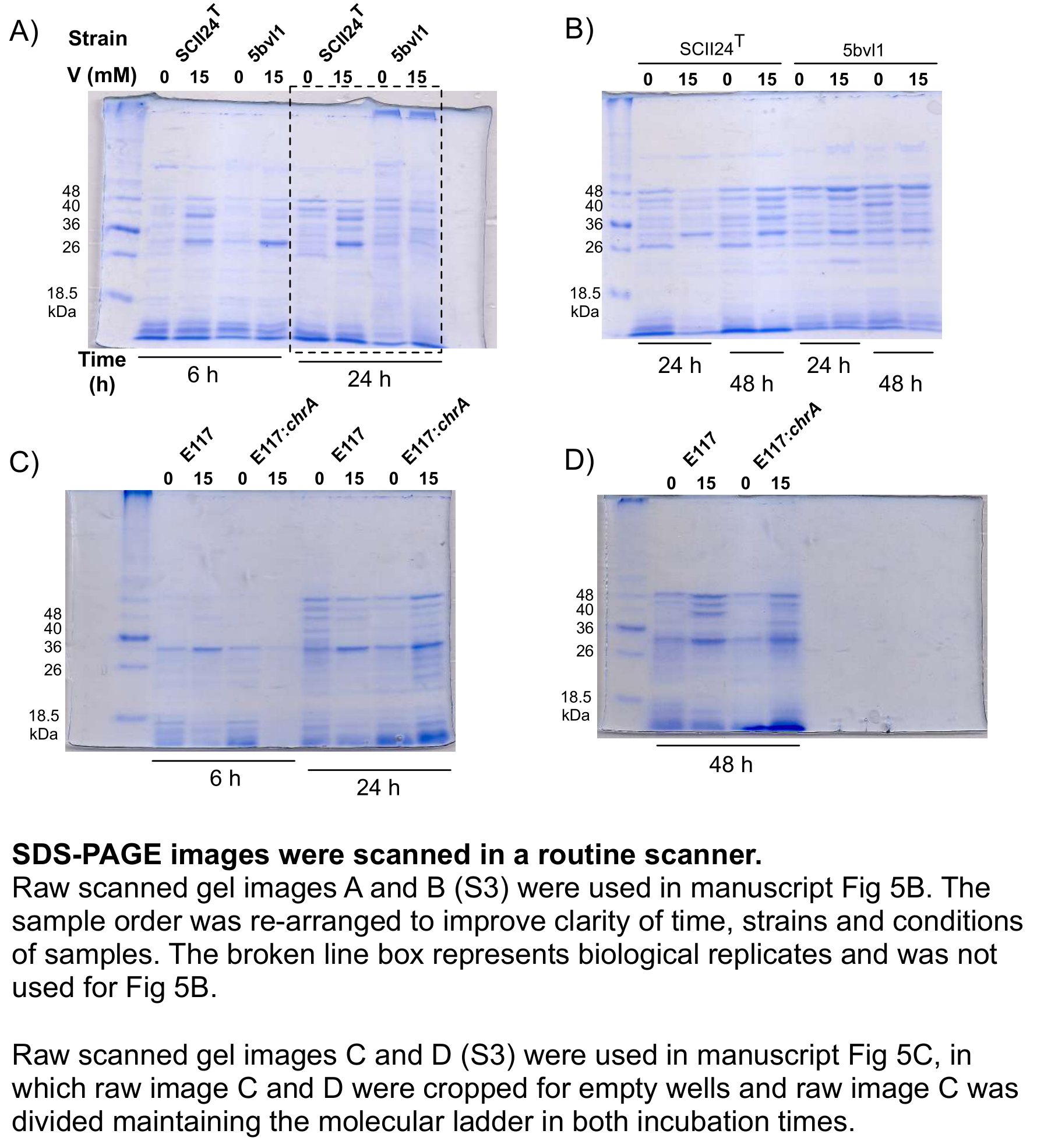

Supplement: S1 Raw images — (TIFF) [file pone.0229359.s003.tiff]
